# Voxelwise-based Brain Function Network using Multi-Graph Model

**DOI:** 10.1038/s41598-018-36155-z

**Published:** 2018-12-10

**Authors:** Zhongyang Wang, Junchang Xin, Xinlei Wang, Zhiqiong Wang, Yue Zhao, Wei Qian

**Affiliations:** 1 School of Computer Science & Engineering, Northeastern University, Shenyang, 110169 China; 2 Sino-Dutch Biomedical & Information Engineering School, Northeastern University, Shenyang, 110169 China; 3 Key Laboratory of Big Data Management and Analytics, Liaoning Province Shenyang, 110169 China; 4 Neusoft Institute of Intelligent Healthcare Technology, Co. Ltd., Shenyang, 110179 China; 5 College of Engineering, The University of Texas at El Paso, Texas, TX 779968 USA

## Abstract

In the research of the fMRI based brain functional network, the pairwise correlation between vertices usually means the similarity between BOLD signals. Our analysis found that the low (0:01–0:06 Hz), intermediate (0:06–0:15 Hz), and high (0:15–0:2 Hz) bands of the BOLD signal are not synchronous. Therefore, this paper presents a voxelwise based multi-frequency band brain functional network model, called Multi-graph brain functional network. First, our analysis found the low-frequency information on the BOLD signal of the brain functional network obscures the other information because of its high intensity. Then, a low-, intermediate-, and high-band brain functional networks were constructed by dividing the BOLD signals. After that, using complex network analysis, we found that different frequency bands have different properties; the modulation in low-frequency is higher than that of the intermediate and high frequency. The power distributions of different frequency bands were also significantly different, and the ‘hub’ vertices under all frequency bands are evenly distributed. Compared to a full-frequency network, the multi-graph model enhances the accuracy of the classification of Alzheimer’s disease.

## Introduction

In recent years, the number of patients suffering from neurodegenerative diseases such as Alzheimer, Parkinson, Mild Cognitive Impairment, and mental disorders such as Depression, Anxiety Neurosis, and Schizophrenia has increased dramatically. Researchers have discovered that using complex network analysis such as small-world^1^, scale-free^2^ and so on can reveal the topological structure of brain functional networks^3–5^. In brain diseases, network topological structures will have different degrees of abnormal changes. Therefore, the study of brain function network based on functional magnetic resonance (fMRI) can not only provide a new perspective on understanding the pathological mechanism of neuropsychiatric diseases but also is helpful for the early diagnosis and treatment evaluation of diseases^6^.

In 2005, Salvador *et al*.^7^ constructed a brain atlas functional network of normal control subjects in a resting state. For the first time, the brain is divided into 90 regions of interest by anatomical automatic labeling (AAL). Most of the later studies of brain function networks have focused on the functional connectivity of brain regions^8–10^. As the atlas-level brain function network takes the mean values of all voxel blood oxygenation level dependent (BOLD) signals in the brain atlas, it will mask the differences in the details between the internal voxels of the atlas. Voxel-wise based brain function network emerges as a more detailed mapping of signals is required^11–13^.

In the study of voxel-wise based functional networks, the pairwise correlation between nodes (vertices) is usually established by using voxel BOLD signal similarity matching in order to obtain correlation matrices of the networks. At present, this similarity is usually a match in the time domain, which is usually matching the full-band information after filtering and removing noise.

In order to increase BOLD weighting, a common feature of most rs-fMRI ICA studies is the use of a relatively long time of repetitions (TRs). The TRs are usually between 2 and 3 s^14^, and the scan durations mostly between 5 and 10 min^15^, limiting the fluctuations that can occur to the frequency ranging between 0.001–0.25 Hz. In most studies, the frequency range is generally controlled between 0.01–0.14 Hz^16^. However, Achard *et al*.^17^ applied discrete wavelet transforms to fMRI time series and they found that in the frequency range of 0.007 to 0.45 Hz, the ‘small-world’ properties of the brain functional networks of different frequency bands all have significant differences. The small-world properties in 0.007–0.01 Hz and 0.23–0.45 Hz are lower than those in other frequency bands, and the small-world properties in 0.01–0.03 Hz and 0.03–0.06 Hz are similar. Compared to the elderly, young people’s brain function network of the frequency band of 0.06–0.1 Hz shows stabler small-world attributes and high local efficiency and global efficiency^18^.

In our experimental analysis, it was found that when the BOLD signal is in the range of 0.06–0.2 Hz, the signal intensity is insignificant when the signal is in the range of 0.06 to 0.15 Hz, and the signal intensity is increased when the signal is greater than 0.15 Hz. Therefore, we split the signal into low (0.01–0.06 Hz), intermediate (0.06–0.15 Hz), and high (0.15–0.2 Hz) frequency bands. Comparing the results of experiment after we split the signal, there are no consistent similarities in different frequency bands. It can be concluded that in the process of brain work, the communication state to vary from with the function and the establishment of a brain functional network model integrating multiple frequency bands will contribute to the study of brain function patterns.

In order to better to describe the similarities and differences between structures of all frequency bands of the brain functional network; this paper proposes a voxel-wise based resting state multi-frequencies brain functional network structure, called the multi-graph brain functional network. Different from the traditional brain network model, a multi-graph-based crossover network model uses the sub-graph under each frequency band as a change of the brain functional network model^19,20^. The acquired voxel BOLD signal is divided into low, intermediate and high frequency bands to keep as much information as possible. The voxels in the fMRI data are used as vertices, and the matching information on vertices in different frequency bands is used as the multi-graph edges to describe the entire model structure. Through analysis, we found that this model can retain more relevant information that can be used to characterize the brain function structure while retaining the nature of the brain network of the previous frequency ranges.

First, our analysis found that the low-frequency information in the BOLD signal of the brain functional network obscures the other information because of its high intensity. Secondly, a low-, intermediate-, and high-band voxel-wise based brain functional network are constructed by the dividing BOLD signals based on frequency. Then, using complex network analysis, we found that different frequency bands have different properties, the comparison shows that the low-frequency and non-frequency-division networks (full frequency band networks) are similar, while the intermediate-high networks have significant differences with non-frequency-division networks. Under different frequency bands, the small-world attributes are well preserved. The low-frequency modulation is higher than that of the intermediate and high frequency, and power distribution of different frequency bands was also significantly different, and the ‘hub’ vertices under all frequency bands are evenly distributed. The multi-graph model can enhance the accuracy of the classification of Alzheimer’s disease compared with no frequency division model.

The contributions of this paper can be summarized as follows.

- The study of brain functional networks using frequency division method found that the BOLD signal similarity matching of voxels in different frequency bands are different.
- A multi-graph-based brain functional network model is proposed. Using the multi-graph model to establish a voxel-wise brain functional network with the combination of low, intermediate, and high-band signals, and a multi-band study of brain function signals is implemented.
- The analysis of the proposed model shows that the topological characteristics of the voxel-wise based brain functional network in the low, intermediate, and high-band signals of the model have obvious differences, which further demonstrates the effectiveness of the crossover network model.

The rest of the paper is organized as follows. In the section: Statistical analysis of pairwise correlation we introduce the experiment and statistics. The details of the proposed multi-graph model are introduced in the section: Multi-graph brain functional network model. The model analysis section shows the model properties which were used in the experiment. Finally, we conclude this paper in the Section: Conclusion.

## Statistical Analysis of Pairwise Correlation

In this section, the data sources and preprocessing is introduced first, and then the similarity of voxel BOLD signals in different frequency bands are discussed.

### Functional magnetic resonance imaging data

#### The experimental data

fMRI data were obtained from the open access NITRC^21^: 1000 Functional Connectomes Project (https://www.nitrc.org/). 662 subjects were used as shown in Table 1. Ethical statements are present in the NITRC, and we confirmed that all experiments were performed in accordance with relevant guidelines and regulations of the access. We also confirmed that all experimental protocols were approved by Sino-Dutch Biomedical & Information Engineering School, Northeastern University, China.

**Table 1:** Experimental data.

Dataset	Abbr.	Subjects	Ages	TR	Slices	Timepoints
Baltimore	Bal	23 [8M/15F]	20–40	2.5	47	123
Bangor	Ban	20 [20M/0F]	19–38	2	34	265
Beijing Zang	BeZ	198 [76M/122F]	18–26	2	33	265
Berlin Margulies	BeM	26 [13M/13F]	23–44	2.3	34	195
Cambridge Buckner	CaB	198 [75M/123F]	18–30	3	47	119
Cleveland	Cle	31 [11M/20F]	24–60	2.8	31	127
Dallas	Dal	24 [12M/12F]	20–71	2	36	115
ICBM	Icb	86 [41M/45F]	19–85	2	23	195
Leipzig	Lei	37 [16M/21F]	20–42	2.3	34	195
Newark	New	19 [9M/10F]	21–39	2	32	135

#### Preprocessing

For each subject, the first 7 volumes of the functional images were discarded for signal equilibrium and to allow the participant to adapt to the experiment, leaving rest volumes for next steps. Standard preprocessing was applied on rs-fMRI dataset of all patients using Data Processing Assistant for Resting State fMRI (DPARSF) toolbox^22^ and Statistical Parametric Mapping Software Package package (*SPM*12) (http://www.fil.ion.ucl.ac.uk/spm)^23^. Slice-timing correction to the last slice was also performed. fMRI time-series realigned using a six-parameter rigid-body spatial transformation to compensate for head movement effects. Then all images were normalized into the Montreal Neurological Institute (MNI) space and re-sampled to 3–*mm* isotropic voxels. A band-pass filtered (0.01–0.2 Hz) was used to retain the relevant information.

#### Pairwise correlation between vertices

In the establishment of a voxel-wise brain functional network, the similarity of voxel BOLD signals is usually used as the pairwise correlation between vertices, and the signal is usually regarded as a full frequency band signal (filtered image). First, Fourier transform is used to transform voxel signals into frequency domain. Second, the frequency domain signal is split into low, intermediate and high ranges. Third, the frequency ranges is restored to the same time domain signal with the same length through inverse Fourier Transform, which is the corresponding frequency band signals, the brain functional networks are established according to the correlation between these separated band signals.

Two voxels in the same frequency band are represented as *x*(*x*_1_, *x*_2_, …, *x*_*n*_) and *y*(*y*_1_, *y*_2_, …, *y*_*n*_). The similarity of the BOLD signal variation sequence is calculated by Euclidean Distance proposed by Muldoon *et al*.^1^, and we can get the correlation by setting the threshold in the equation below.

Euclidean distance between vertex *x* and *y* is defined as

1 $$d(x,\,y)=\sqrt{\sum _{1}^{n}\,{({x}_{n}-{y}_{n})}^{2}}$$

When the *d*(*x*, *y*) is smaller than the threshold *d*(*x*, *y*) <*λ*, then we can say that there is a functional connection, i.e. the pairwise correlation.

### Statistical analysis of similarity of BOLD signal in different frequency bands

**Figure 1:**
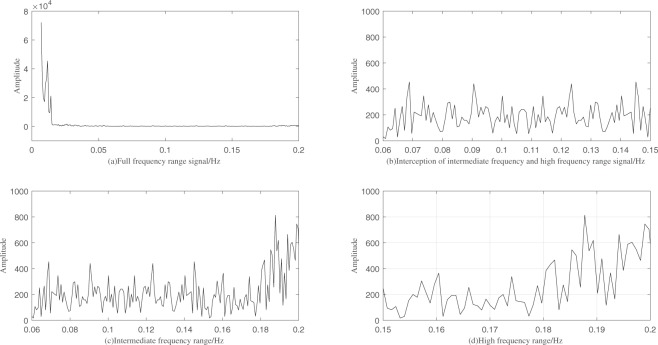
Typical examples BOLD signal decomposed. (**a**) BOLD signal in frequency domain (0.01–0.2 Hz). (**b**) BOLD signal interception by removal of low frequency range (0.06–0.2 Hz). (**c**) Intermediate frequency interception range signal (0.06–0.15 Hz). (**d**) High frequency interception range signal (0.15–0.2 Hz).

In the study of BOLD signal in brain voxel, the low frequency range shows a very high signal intensity, which is difficult to see in other frequency domain, displayed in Fig. 1(a). When the low frequency range is removed, and when the signal is larger than 0.15 Hz, the waveform has an upward trend in the interception signal ranges, shown in Fig. 1(b). in the study of Achard^17^, the small world properties in 0.01–0.06 Hz frequency are similar, and the small world properties in 0.06–0.23 Hz frequency are falling, and the changes in the amplitude around 0.06–0.15 Hz and 0.15–0.2 Hz are different from our experiments, as shown in Fig. 1(c,d).

**Figure 2:**
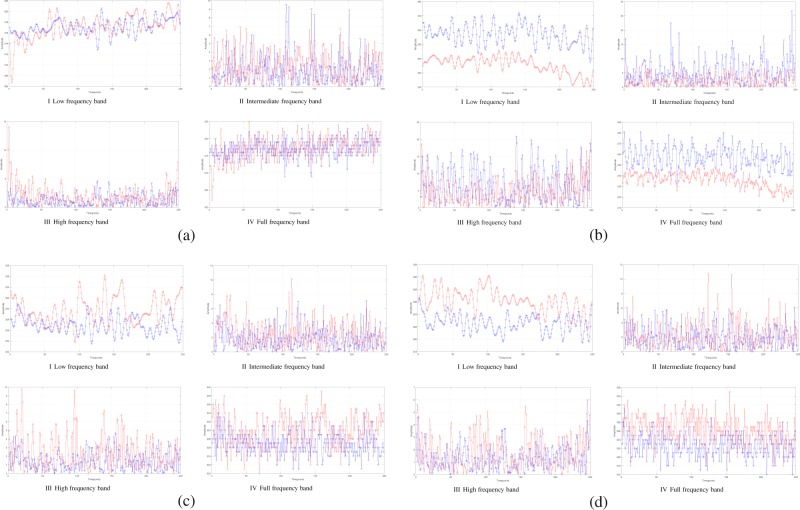
Two voxels with different signal patterns are sampled by the voxel’s BOLD signal of brain network, which are represented by red and blue lines respectively. (**a**) Voxel signals are similar in low, intermediate, high frequency band, and are similar in all frequency bands. (**b**) Voxel signals are similar in low, intermediate and high frequency band, and are dissimilar in full frequency band. (**c**) Voxel signals are dissimilar in low, intermediate and high frequency band, and are similar in full frequency band. (**d**) Voxel signals are dissimilar in low frequency band, and are similar in the intermediate, high frequency and full frequency band.

Since there are obvious differences in different frequency ranges, it is necessary to study the differences of BOLD signals in the frequency bands. We found that there are many situations that are similar in a matching of functional signals on different frequency bands. The voxels with different signal situations were sampled by the voxel’s BOLD signal of brain network. In each situation, the two voxels were represented by red and blue in Fig. 2. As shown in Figure 2(a)I–III, signal matches in the full frequency band signal, when the two voxels of brain are matched at low, intermediate and high frequencies respectively. We can also see that the signal also matches in the full frequency band, that is shown in Fig. 2(a)IV.

Meanwhile, we also found that some voxels have another state, as shown in Fig. 2(b), two BOLD signals are not similar in the full frequency band. However, after the signals are separated by low, intermediate and high frequency band, their signals similar in all three bands. We also found that when some signals are similar in the full frequency band, the signals are not similar at low, intermediate and high frequency bands and results are shown in Fig. 2(c). In the study, there are also quite a few signals which are not similar under the low frequency band, but are similar in intermediate and high frequency band, the results show high similarity, and it also shows similarity in full frequency band signal as shown in Fig. 2(d).

Therefore, it is necessary to consider the effect of this BOLD signals similarity difference on the pairwise correlation in functional networks. In order to summarize and analyze the problems found in the experiment, the brain functional networks about 14000 vertices are established at low, intermediate, high frequencies and full frequency bands respectively under the same threshold *λ*. The low frequency network correlation matrice is represented as *H*_*L*_, the intermediate frequency network is represented as *H*_*I*_, the high frequency network is represented as *H*_*H*_, and the full frequency bands network is represented as *H*_*Full*_.

The results displayed in the Table 2 are the number of pairwise correlations (i.e. edges) between vertices in the brain network mentioned above. First, as shown from rows 1 to 4 in Table 2, the edges contained in *H*_*L*_, *H*_*I*_ and *H*_*H*_ is different from *H*_*Full*_, which shows the difference of connectivity patterns in different frequency bands. Secondly, from rows 5–15, we can determine that there is a large number of overlaps at the edges of the network in different frequency bands. Especially when compared with *H*_*Full*_, *H*_*L*_ covers 96.91% of the edges of *H*_*Full*_. The intersection ratios of *H*_*I*_ and *H*_*H*_ are 13.18% and 15.63% respectively. From the results above, we can determine that the impact of low frequency information of the signal in *H*_*Full*_ is more obvious.

**Table 2:** The comparative analysis of low frequency band network *H*_*L*_, intermediate frequency band network *H*_*I*_, high frequency band network *H*_*H*_ and full frequency band network *H*_*Full*_.

Label	Correlation Matrices	Pairwise Correlations
1	|*E* (*H*_*L*_)|	8,987,853
2	|*E* (*H*_*I*_)|	12,320,070
3	|*E* (*H*_*H*_)|	12,664,665
4	|*E* (*H*_*Full*_)|	9,134,745
5	|*E* (*H*_*L*_ ∩ *H*_*I*_)|	1,119,440
6	|*E* (*H*_*L*_ ∩ *H*_*H*_)|	1,382,865
7	|*E* (*H*_*I*_ ∩ *H*_*H*_)|	2,702,296
8	|*E* (*H*_*L*_ ∩ *H*_*Full*_)|	8,852,743
9	|*E* (*H*_*L*_ ∩ *H*_*Full*_)|	1,204,037
10	|*E* (*H*_*H*_ ∩ *H*_*Full*_)|	1,427,283
11	|*E* (*H*_*L*_ ∩ *H*_*I*_ ∩ *H*_*Full*_)|	1,118,700
12	|*E* (*H*_*L*_ ∩ *H*_*H*_ ∩ *H*_*Full*_)|	1,379,541
13	|*E* (*H*_*I*_ ∩ *H*_*H*_ ∩ *H*_*Full*_)|	323,659
14	|*E* (*H*_*L*_ ∩ *H*_*I*_ ∩ *H*_*H*_)|	304,544
15	|*E* (*H*_*L*_ ∩ *H*_*I*_ ∩ *H*_*H*_ ∩ *H*_*Full*_)|	304,481
16	|*E* (*H*_*L*_ ∪ *H*_*I*_)|	20,188,483
17	|*E* (*H*_*L*_ ∪*H* _*H*_)|	20,269,653
18	|*E* (*H*_*I*_ ∪ *H*_*H*_)|	22,282,439
19	|*E* (*H*_*L*_ ∪ *H*_*I*_ ∪ *H*_*H*_)|	29,072,531
20	|*E* (*H*_*L*_ ∪ *H*_*I*_ ∩ *H*_*Full*_)|	8,938,080
21	|*E* (*H*_*L*_ ∪ *H*_*H*_ ∩ *H*_*Full*_)|	8,900,485
22	|*E* (*H*_*I*_ ∪ *H*_*H*_ ∩ *H*_*Full*_)|	2,307,661
23	|*E* (*H*_*L*_ ∪ *H*_*I*_ ∪ *H*_*H*_ ∩ *H*_*Full*_)|	8,966,644

When we assemble different frequency bands together, the coverage ratio is also different. The intersection ratio of *H*_*L*_ and *H*_*I*_ covers 12.24% of *H*_*Full*_, and intersection ratio of *H*_*L*_ and *H*_*H*_ is 15.1%. These two situations are higher than those of *H*_*I*_ and *H*_*H*_, who has an intersection ratio of 3.54%. It can be seen that the influence of high frequency signal on the full frequency band signal is higher than intermediate frequency signal on full frequency band signal.

As indicated in |*E*(*H*_*L*_ ∩ *H*_*I*_ ∩ *H*_*Full*_)|, |*E*(*H*_*L*_ ∩ *H*_*H*_ ∩ *H*_*Full*_)| and |*E*(*H*_*I*_ ∩ *H*_*H*_ ∩ *H*_*Full*_)|, most of the edges in the intersection |*E*(*H*_*L*_ ∩ *H*_*I*_)| and |*E*(*H*_*L*_ ∩ *H*_*H*_)| of *H*_*I*_, *H*_*L*_ and *H*_*H*_ exist in *H*_*Full*_. But there is only 11.98% edges of the intersection |*E*(*H*_*I*_ ∩ *H*_*H*_)| belong to *H*_*Full*_. You can see that the low and high frequency components are the main factors affecting *H*_*Full*_.

As shown in Table 2 from 16–23, after adopting the union of two or more than two kinds of frequency bands for analysis, it can be reveal that the union of *H*_*L*_ and *H*_*I*_, as |*E*(*H*_*L*_ ∪ *H*_*I*_)|, covers up to 97.85% information of *H*_*Full*_, and other union such as |*E*(*H*_*L*_ ∪ *H*_*H*_)| can cover 97.44% of *H*_*Full*_. While the union of three bands can cover 98.15% of the *H*_*Full*_ information. The union of *H*_*I*_ and *H*_*H*_ covers less information. Only 25.26%. At the same time, it can be seen from the Table 2, a lot of edges cannot be displayed in the *H*_*L*_, *H*_*I*_, *H*_*H*_ network. Therefore, due to the coverage of low frequency information, the use of full band frequency signals to match nodes will lose a large amount of information.

The use of *p*-values is common in statistical hypothesis testing for significance, the *p*-values are the probability, reflecting the probability of an event. According to the *p*-values obtained by the significance test method, *p* > 0.05 is generally considered to be no statistically significant differences, *p* < 0.05 is generally considered to be statistically significant differences, *p* < 0.01 is considered to be extremely statistically significant differences. Therefore, we use *p*-values to calculate whether there is a significant difference between networks with different frequency bands. In summary, the smaller the *p*-values, the more significant the result. From Table 3, the differences between *H*_*L*_ and *H*_*Full*_ are not obvious, as the *p*-value is 0.0672. And by the *p* around 0.01, *H*_*L*_ differs significantly from *H*_*I*_ and *H*_*H*_. There is also a significant difference between *H*_*I*_ and *H*_*H*_, but it is lower than *H*_*L*_.

**Table 3:** The statistics difference significance test by *p*-value of networks.

*p*-value	*H*_*L*_	*H*_*I*_	*H*_*H*_	*H*_*Full*_
*H*_*L*_	—	0.0158	0.0094	0.0672
*H*_*I*_	0.0158	—	0.0476	0.0149
*H*_*H*_	0.0094	0.0476	—	0.0283
*H*_*Full*_	0.0672	0.0149	0.0283	—

## Multi-Graph Brain Functional Network Model

### Multi-graph network model

In order to describe the brain network better, a voxel-wise based brain crossover functional network by the multi-graph model is proposed in this paper, in which the low, intermediate and high frequency information is integrated. The matching information between vertices is described by multiple edges, which form the network model by multi-graph. According to the different working modes, the number of edges between different vertices may also be different.

The multi-graph network model is represented as a network model *G* = (*V*, *E*), *V*(*G*) represents the set of vertices with all voxels. *E*(*G*) represents the set of edges in *G*. As *E*(*G*) = E_*L*_ ∪ E_*I*_ ∪ E_*H*_, E_*L*_, E_*I*_, E_*H*_ are expressed as a combination of low, intermediate and high frequency edges. In this model, the connection relation between any two vertices can be described by three modes: low, intermediate and high frequency model. The edges between two vertices *i* and *j* are *e*_*ij*_ = {*e*_*Lij*_, *e*_*Iij*_, *e*_*Hij*_}.

Under this model, *H* is defined as a subgraph of *G*. If *V*(*H*) = *V*(*G*), and *E*(*H*) ⊆ *E*(*G*), then Let *k* be any positive integer. Let

2 $$G={H}_{1}\cup {H}_{2}\cup \cdots \cup {H}_{{\rm{k}}}$$

Namely *G* will be decomposed into *k* subgraphs, so that *H*_1_, *H*_2_, …, *H*_k_ are the subgraphs of *G*. *H*_1_, *H*_2_, …, *H*_k_ are pairwise edge disjoint. Using this method we can realize the full coverage of the brain network, and can also decompose the multiple possibilities of the brain network operating mode. *H*_1_, *H*_2_, …, *H*_k_ can be used to represent different brain activities.

There are two patterns of work when studying the connectivity of brain network vertices.

**Figure 3:**
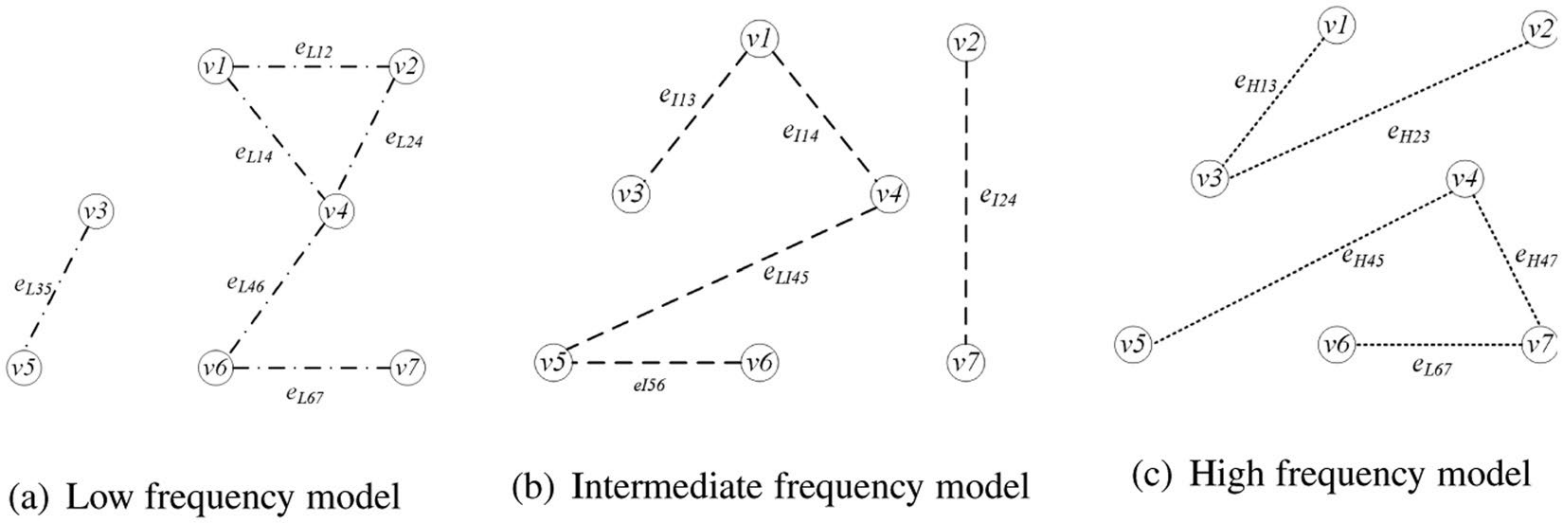
Example of first connection pattern.

First pattern is the separation of low, intermediate and high frequency. If *e*_*ij*_ = *e*_*Lij*_, the network is a low frequency network model. The brain function network model is expressed as *H*_*L*_. Similarly, when *e*_*ij*_ = *e*_*Iij*_ or *e*_*ij*_ = *e*_*Hij*_, the intermediate and high frequency network model is *H*_*I*_, *H*_*H*_. Examples are shown in Fig. 3. Figure 3(a) is the low frequency model, the edges between vertices are low frequency bands. Figure 3(b) is the intermediate frequency model, the edges between vertices are intermediate frequency bands. Figure 3(c) is the high frequency model, the edges between vertices are high frequency bands.

**Figure 4:**
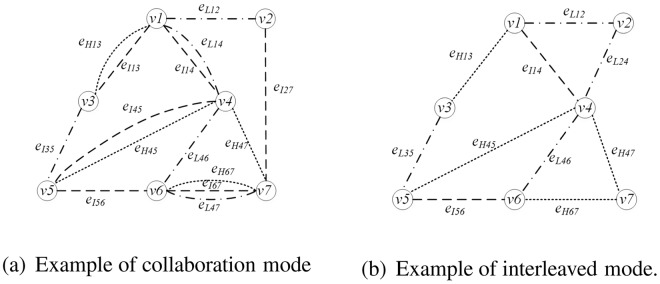
Example of second connection pattern.

Second pattern is the mixed pattern of low, intermediate and high frequency. There are two attended model. First, when there are two or more connections in *e*_*Lij*_, *e*_*Iij*_, *e*_*Hij*_, it can be considered as edges existing between two nodes. Examples are *e*_*ij*_ = *e*_*Lij*_, *e*_*Iij*_, *e*_*ij*_ = *e*_*Lij*_, *e*_*Hij*_, *e*_*ij*_ = *e*_*Iij*_, *e*_*Hij*_ and *e*_*ij*_ = *e*_*Lij*_, *e*_*Iij*_, *e*_*Hij*_, which is called collaboration model. Therefore, low- intermediate frequency, low-high frequency, intermediate-high frequency and low-intermediate-high frequency models are expressed as *H*_*LI*_, *H*_*LH*_, *H*_*IH*_, *H*_*LIH*_. Second, if there is any edge in *e*_*Lij*_, *e*_*Iij*_, *e*_*Hij*_, then it is considered an edge between two vertices, which are *e*_*ij*_ = {*e*_*Lij*_, *e*_*Iij*_, *e*_*Hij*_}. This work pattern is represented as ${H^{\prime} }_{LIH}$, which called interleaved model. Examples are shown in the Fig. 4. Figure 4(a) is the example of collaboration model, there are many edges between vertices in the graph. Figure 4(b) is the example of interleaved model, edges with different bands exist simultaneously between vertices in the graph.

### Multi-graph model analysis

According to the multi-graph model mentioned above, *H*_1_, *H*_2_, …, *H*_k_ can be used to represent different brain activities, whose organization forms represent different brain network models. Therefore, the proposed multi-graph network model based on low, intermediate and high frequency bands *H*_*L*_, *H*_*I*_, *H*_*H*_ is discussed with full frequency band signal *H*_*Full*_.

#### Small-world properties

We can see from the existing research, the brain functional network has small-world properties. Therefore, the small world attributes of the low, intermediate and high frequency network will need to be analyzed^1^. In the analysis of small world networks, there are generally two parameters: Clustering coefficient and the shortest path. The small world property of human brain network has important function meaning.

**Figure 5:**
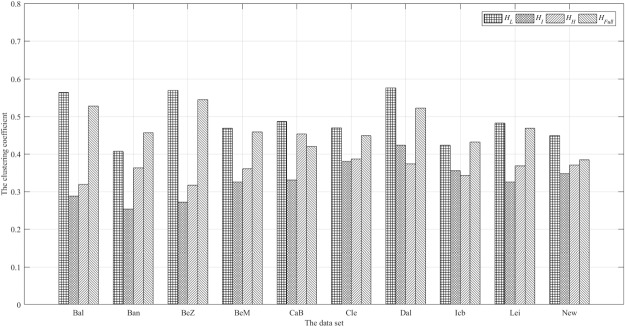
The clustering coefficient ‘*C*’: ${C}_{{H}_{L}}$, ${C}_{{H}_{I}}$, ${C}_{{H}_{H}}$, ${C}_{{H}_{Full}}$.
- The shortest path
  The shortest path length *L* represents the number of edges required by communicating between any two vertices in the network^1^. The length of the shortest path means that there is a fast and efficient information transfers function between the cerebral regions within the network, and the ability to transfer information fast lays the foundation for the functional integration of the network. The average shortest paths are shown in Fig. 6.

- Clustering coefficient
  According to the graph theory, clustering coefficient *C* is a coefficient of the vertices aggregation degree in a graph^1^. Clustering coefficient means separation module, that is, the network has the ability of efficient modular processing of information. The network that has a higher average clustering coefficient was found to have a modular structure in the study. The calculation results are shown in Fig. 5.

**Figure 6:**
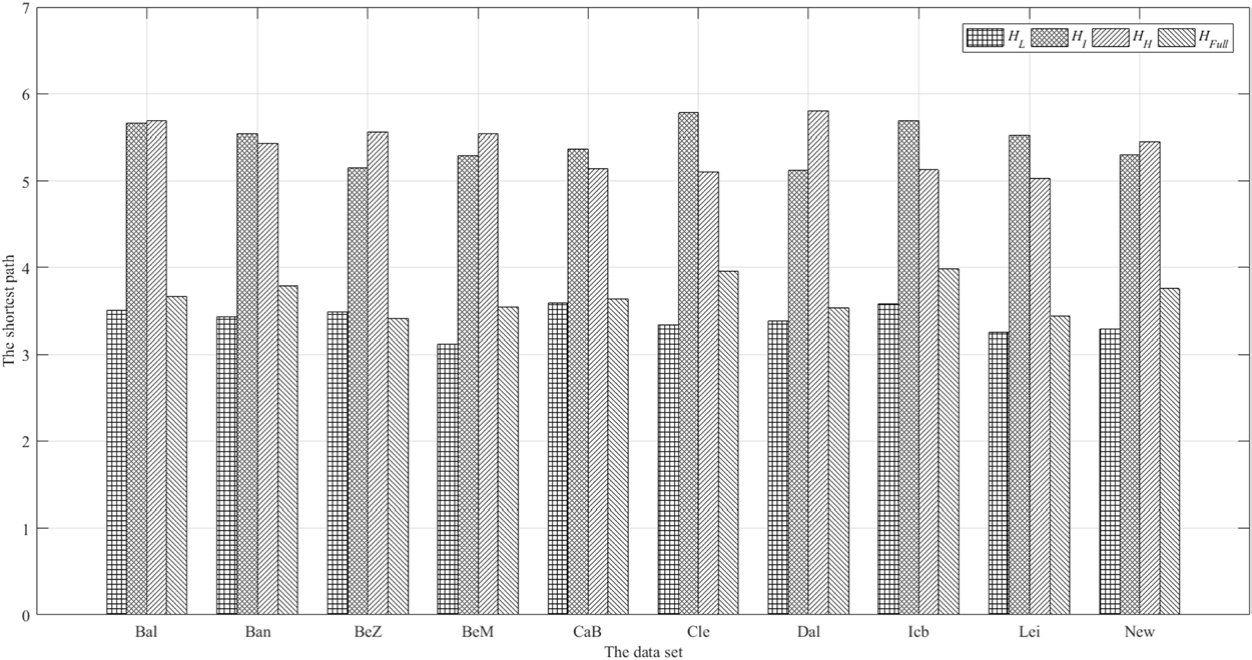
The shortest path ‘*L*’: ${L}_{{H}_{L}}$, ${L}_{{H}_{I}}$, ${L}_{{H}_{H}}$, ${L}_{{H}_{Full}}$.
- Small-world analysis summary

To diagnose small-world properties, the characteristic path length and clustering coefficient were compared with the same metrics estimated in random networks configured with the same number of vertices, mean degree, degree distribution as the network of interest. This network is expressed as ${H}_{L}^{R}$, ${H}_{I}^{R}$, ${H}_{H}^{R}$. The clustering coefficient is expressed as ${C}_{L}^{R}$, ${C}_{I}^{R}$, ${C}_{H}^{R}$. The shortest path is expressed as ${L}_{L}^{R}$, ${L}_{I}^{R}$, ${L}_{H}^{R}$. Typically, in a small-world network, we expect the ratio *γ* = *C*/*C*^*R*^ > 1 and the ratio *λ* = *L*/*L*^*R*^ > 1. A scalar summary of small-worldness is therefore the ratio *σ* = *γ*/*λ*, which is typically >1.

**Figure 7:**
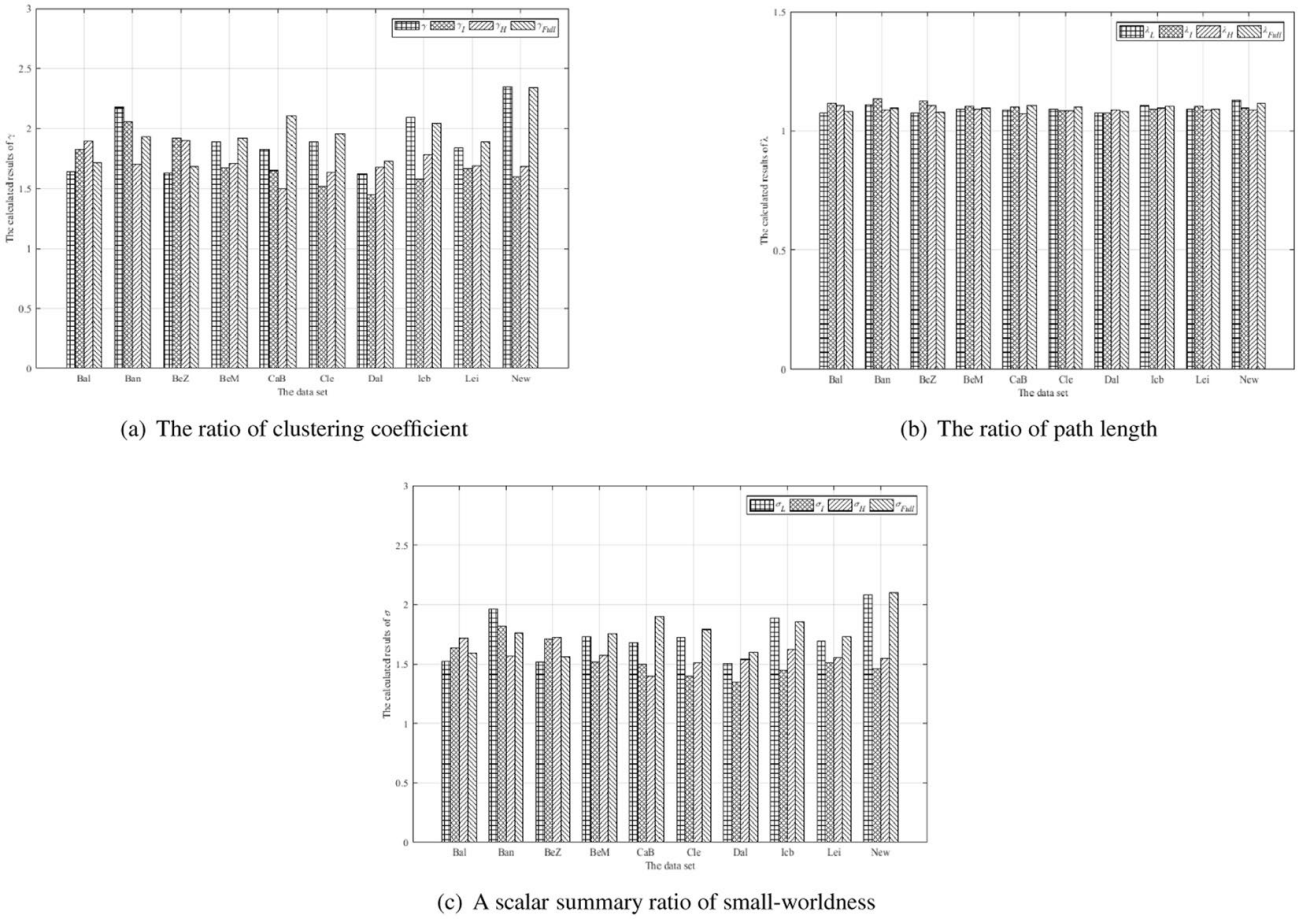
The ratio of clustering coefficient, path length and small-worldness.

It can be seen that all subgraphs retain better small world properties under voxel-wise network, as shown in Fig. 7.

First, as shown in Figs 5 and 7(a), the low frequency subgraph *H*_*L*_ has a higher clustering coefficient than others. Due to the higher clustering coefficient, the node connection appears a higher degree of modular distribution, which means that brain function network mainly forms a modular structure to complete the work under the subgraph *H*_*L*_. The modular connectivity of other subgraphs is less than *H*_*L*_.

Second, the subgraph *H*_*I*_ and *H*_*H*_ have a lower clustering coefficient than the subgraph *H*_*L*_, which means that the modular distribution degree of *H*_*I*_ and *H*_*H*_ is lower than that of the subgraph *H*_*I*_. Thus it can be seen that the network structures are more global under the subgraph *H*_*I*_ and *H*_*H*_. The working subgraph of *H*_*I*_ and *H*_*H*_ will tend to be the cooperative model of whole brain vertices.

Finally, the *H*_*I*_ has a similar clustering coefficient with the *H*_*ALL*_, which is due to the strong low-frequency signal, overlapping other signals.

From the Fig. 7(b) the length of the shortest path of the network varies with different network structures. At the same time of modular communication in the brain, the vertices maintain a high degree of connectivity, proving that the brain network is an efficient and integrated whole. Therefore, as shown in Fig. 7(c), we can see that low, intermediate and high frequency networks and their combinations have small-world properties.

#### Scale-free analysis

**Figure 8:**
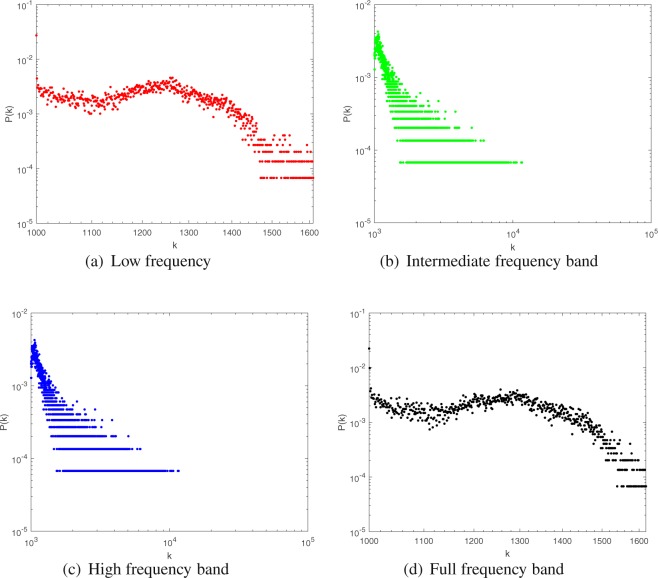
Scale free statistical comparison.

Due to the discussion of scale-free properties of brain networks in existing researches, scale-free networks have serious heterogeneity. In addition, the connection condition (degree) of each vertex in scale-free network has serious uneven distribution: In the network, a few vertices, called Hub vertices, have vast connections, and most nodes only have a small number of connections. Therefore, in this paper, the scale-free properties of low, intermediate, high frequency band and full frequency band are statistically analyzed. As the results of all data sets are similar, only the ‘Cambridge Buckner’ data-set is used as an example for subsequent experiments and the results are shown in Fig. 8.

The statistics of the power rate characteristics in low, intermediate, high frequency and full frequency band network can be seen from the results. During the analysis of scale-free properties of the proposed network, the scale free curves shown in full frequency band are similar to those shown in low frequency band, but are not strict scale free curves. In the intermediate and high frequency, we can determine that the structure has scale-free characteristics, the low-frequency working state of the brain is more prominent, and that the intermediate and high frequency band also contains a lot of relevant information. Therefore, in the voxel level multi-graph model, the brain network exhibits some scale-free properties.

Similarly, we use the *p*-value to calculate the significant differences under different frequency bands. As shown in Table 4, the degree distributions of scale-free analysis between *H*_*L*_, *H*_*I*_, *H*_*H*_, and *H*_*Full*_ are differences. The *H*_*L*_ has the significant differences from *H*_*I*_ and *H*_*H*_, but there is no significant differences between *H*_*L*_ and *H*_*Full*_. And the *p*-value between *H*_*I*_ and *H*_*H*_ is 0.0773, which shown no difference. Therefore, it can be seen that, through the statistical test, low frequency and full frequency band have similar degree distribution, intermediate frequency and high frequency band have similar degree distribution.

**Table 4:** The statistics difference significance test by *p*-value on degree distribution of scale-free analysis.

*p*-value	*H*_*L*_	*H*_*I*_	*H*_*H*_	*H*_*Full*_
*H*_*L*_	—	0.0267	0.0325	0.0761
*H*_*I*_	0.0267	—	0.0773	0.0278
*H*_*H*_	0.0325	0.0773	—	0.0302
*H*_*Full*_	0.0761	0.0278	0.0302	—

#### The ‘hub’ vertices

Due to the voxel network structure defined in the study, the vertices cover all voxels in the whole brain. Therefore, it is necessary to analyze the distribution of important vertices (i.e. the ‘hubs’)^24,25^. In order to calculate the importance of vertices, the degree centrality is adopted. Degree centrality is the most direct measure of vertex centrality in network analysis. The greater the vertex degree of vertices, the higher the degree centrality of the vertex, therefore, the vertex is more important in the network.

**Figure 9:**
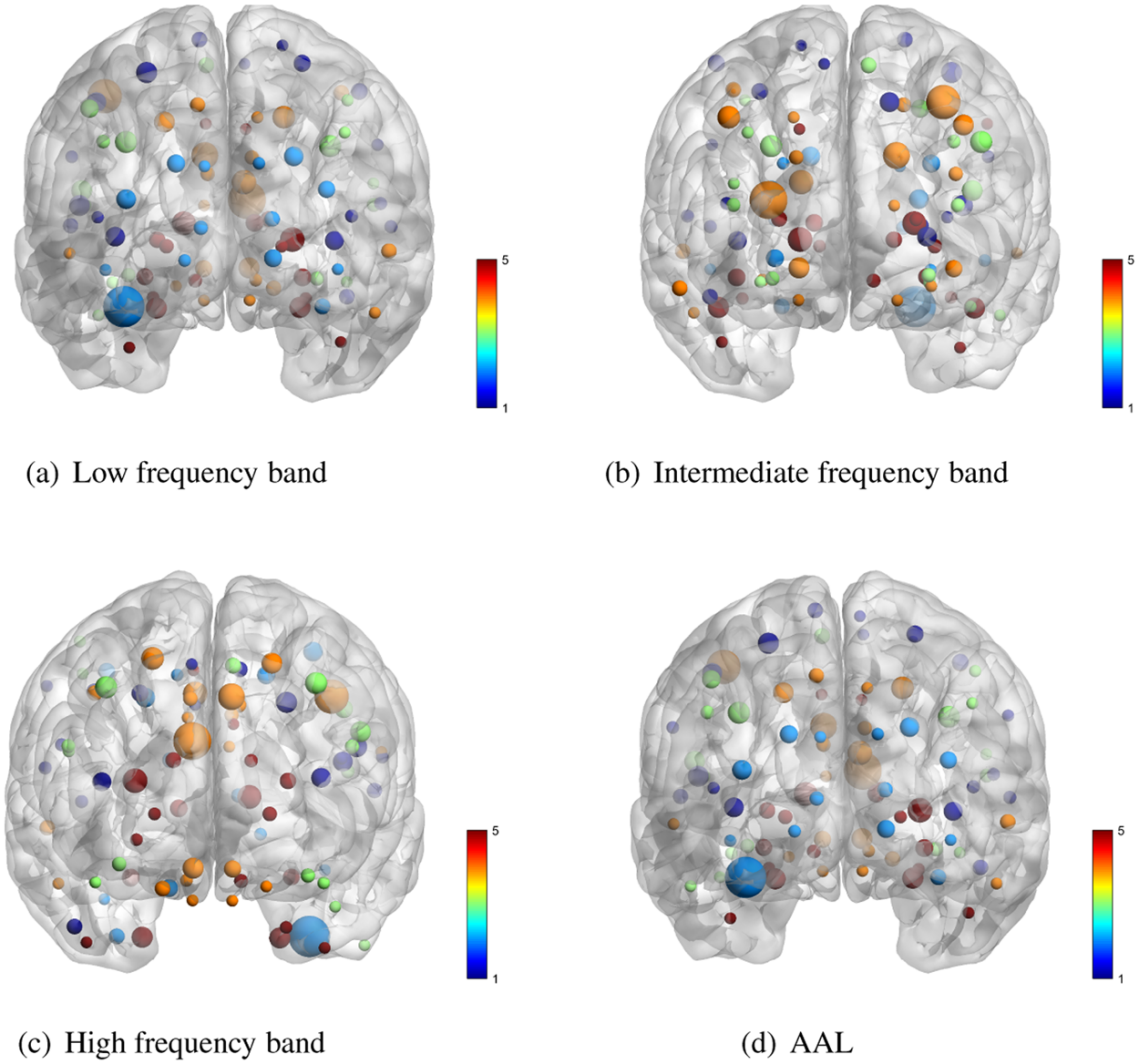
‘Hub’ vertices distribution comparison.

In actual research, the most commonly used vertices distribution is the prior brain automated anatomical labeling (AAL) distribution structure of 90 cerebral regions used by Salvador^7,26,27^. Therefore, after extracting the vertices with the highest degree centrality as the brain network ‘hub’ vertices in *H*_*L*_, *H*_*I*_, *H*_*H*_, we compare the distribution data with AAL as shown in Fig. 9.

**Figure 10:**
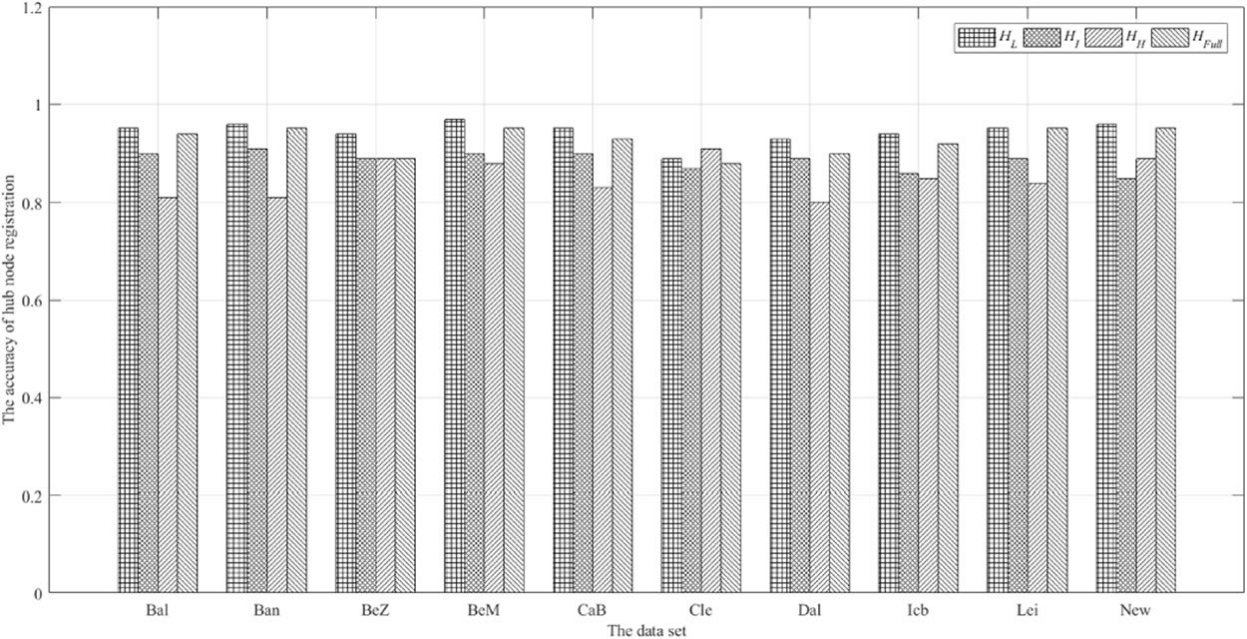
The ‘Hub’ vertices distribution comparison.

In order to show the difference of vertices distribution more intuitively, the distance between the vertex *i* in the obtained network and the central nvertex *k* in the one of regions of AAL is expressed as *d*_*ik*_. Setting the threshold *δ*. If *d*_*ik*_ < *δ*, The node *i* belongs to the cerebral region *k*. If *d*_*ik*_ > *δ*, vertex *i* continue to compare with other cerebral regions. If there are *i*, *j* vertices, and *d*_*ik*_ < *δ*, *d*_*jk*_ < *δ*, then only one count is retained. After all vertices are compared, the total number of vertices matching AAL is represented as *M*. The accuracy of the comparison is expressed as *A* = *M*/90. Calculating A for *H*_*L*_, *H*_*I*_, *H*_*H*_ respectively. The calculation results are shown in Fig. 10.

As seen from the Fig. 10, in the *H*_*L*_, *H*_*I*_ and *H*_*H*_, the calculated vertices can cover all the anatomical cerebral regions. Among them, the cerebral region of hub vertex is the most dispersed in *H*_*L*_, which is dispersed in different cerebral regions, and the vertices in *H*_*I*_ and *H*_*H*_ are second. In different frequency bands, the change of the working model of the brain network leads to the difference of the position of the central ‘hub’ vertex. This shows that nodes tend to work globally in low frequency networks and work more via inter-regional communication in intermediate frequency and high frequency networks.

#### Vertices connection distribution

**Figure 11:**
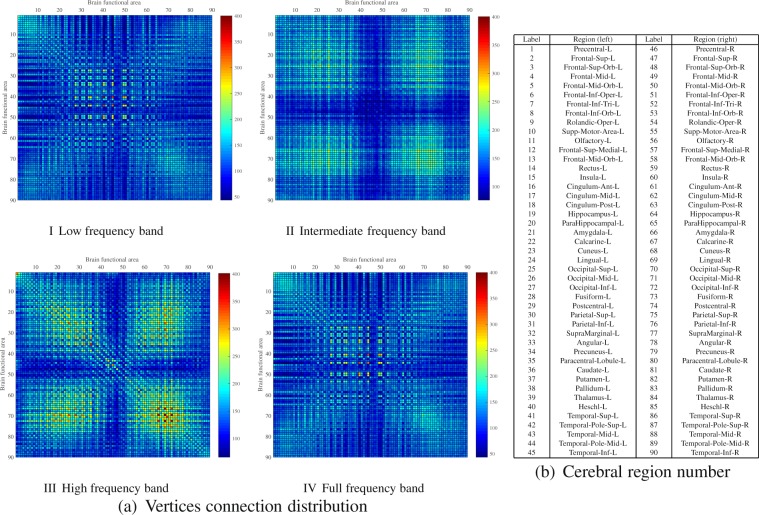
Vertices connection distribution comparison. (**a**) Vertices connection distribution of 90 cerebral region. I: Low frequencies; II: Intermediate frequency; III: High frequency; IV: Full frequency. (**b**) Cerebral region checklist table.

In order to investigate the connection patterns among vertices, the connectivity patterns of all vertices in the whole brain are statistically analyzed. First, all vertices are registered into 90 cerebral region of AAL^26^. All the vertices are clustered according to the cerebral region. Next, count the number of connection edges between the vertices in each cerebral region and the vertices in all other cerebral regions. The brightness of each lattice in the matrix reflects the number of relationships between the two cerebral regions, shown in Fig. 11(a). The cerebral region corresponding to the number is shown in Fig. 11(b), label 1–45 represent the left portion of the brain, and label 46–90 represent the right portion of the brain.

The vertices connection distribution matrixes are also test by *p*-value, as shown in Table 5. According to the table, the vertices connection distribution on full frequency band has no significant difference with the low frequency band by the *p* = 0.0679. and low-, intermediate-, and high frequency band have the significant differences as the *p* < 0.05.

**Table 5:** The statistics difference significance test by *p*-value on vertices connection distribution.

*p*-value	*H*_*L*_	*H*_*I*_	*H*_*H*_	*H*_*Full*_
*H*_*L*_	—	0.0312	0.0285	0.0679
*H*_*L*_	0.0312	—	0.0436	0.0334
*H*_*L*_	0.0285	0.0436	—	0.0452
*H*_*L*_	0.0679	0.0334	0.0452	—

As shown in Fig. 11(a)I, in the low frequency brain function network, the connectivity of the vertices in the cerebral region shows a uniform state. Previous studies have shown that cerebral regions show a higher degree of correlation at low frequencies^28^. Therefore, it can be said that in low frequency brain functional networks, the connections between different regions of the brain are mainly low-frequency connections, and this connections are similar to that of Fig. 11(a)IV. We can concluded that in the whole brain, low frequency connection is more significant in the remote cerebral region connection. But in the diagonal dominated region in Fig. 11(a)I, the connectivity is stronger. This situation is more obvious in Fig. 11(a)II,III. By comparing Fig. 11(a)I,IV with Fig. 11(a)III and Fig. 11(a)III, low frequency and full frequency band functional network show high similarity. Therefore, the brain network constructed with full frequency band covers a large amount of information in Fig. 11(a)III and Fig. 11(a)III.

By observing Fig. 11(a)II,III, in the intermediate frequency and high frequency brain functional networks, the connectivity of the brain network shows a higher local connectivity, which cannot be expressed in the full frequency band signal or low frequency signal. In Fig. 11(a)III, we can see that the connectivity structure of the cerebral region on the diagonal presents a state of high brightness. In the network, the connectivity of the brain is dominated by its own internal communications and the proximity of cerebral regions this can be seen from the top right of Fig. 11(a)III. The left cerebral region in the brain corresponds to the right cerebral region in a highly connected state, which is due to the same or similar function of the left and right corresponding areas in the original AAL partition. This situation, as shown in Fig. 11(a)II, is also reflected in the network. From Fig. 11(a)II,III, we can see the left and right brain connections show a high intensity distribution on the diagonal on the lower left region of the image. It can be concluded that the functional connection between the cerebral region, the adjacent cerebral region and the left and right brain is mainly connected by intermediate and high frequency bands.

Therefore, the correlation between voxels and the function of brain regions are taken into account, if only the full frequency band signal is used as the main signal of brain network analysis, then a considerable amount of information in the research of brain function network will be covered by low frequency signals. Therefore, when establishing the brain functional network model, the low frequency, intermediate frequency and high frequency information separation analysis can be used to create a larger, multi-pattern network model, which contributes significantly towards the study of brain function.

#### Multi-graph network based Alzheimer’s disease classification

Brain network alterations in patients with Alzheimer’s disease (AD) has been the subject of much research, but the biological mechanisms underlying these alterations remain poorly understood^29,30^. In this section, we verify whether the multi-graph model can be used for classification of these patients from healthy control (HC) subjects and whether we can achieve better classification accuracy than the no frequency division model.

In typical connectivity-networks-based classification approaches, local measures of connectivity networks are first extracted from each region-of-interest as network features, which are then concatenated into a vector for subsequent feature selection and classification. However, some useful structural information of network, especially global topological information, may be lost in this type of approaches. To address this issue, Jie *et al*.^31^ proposed a connectivity-networks-based Alzheimer’s classification framework that involves the use of a new recursive feature elimination method based on graph kernel to measure directly the topological similarity between connectivity networks and use kernel Support Vector Machine (k-SVM) to fuse all features from multiple thresholded networks for final classification (namely, RFE-GK).

Jie *et al*.^31^ has already demonstrated the advantage of RFE-GK in the classification of Alzheimer’s disease compared with other methods. And all the Alzheimer’s disease classification methods (including RFE-GK) in Jie’s work are still based on the non-frequency division network model (i.e. Full frequency band network). Therefore we only compared the proposed multi-graph model with the RFE-GK.

For the multi-graph model, we also use the method of graph kernel construction proposed by Jie *et al*.^31^. However, the difference is that the graph kernel we built is based on the multi-graph model. If there are *n* subjects (including AD and HC), for the subgraph ${\chi }_{i}^{m}$ of the subject *i* under the frequency band *m*, the graph kernel is expressed as

3 $$Kerne{l}_{i}^{m}=(f({\chi }_{i}^{m},\,{\chi }_{1}^{m}),\,f({\chi }_{i}^{m},\,{\chi }_{2}^{m}),\,\ldots f({\chi }_{i}^{m},\,{\chi }_{j}^{m}),\,f({\chi }_{i}^{m},\,{\chi }_{n}^{m}))$$

where, $f({\chi }_{i}^{m},\,{\chi }_{j}^{m})$ is the similarity between subjects *i* and subjects *j*, $Kerne{l}_{i}^{m}$ represents the graph kernel of subject *i*.

Then a fusion graph kernel is learned through the linear combination of the graph kernels in the all *k* band.

4 $$Kernel=\sum _{m=1}^{{\rm{k}}}\,{\mu }_{m}Kerne{l}_{i}^{m}$$

where, *μ*_*m*_ is a non negative weight vector and satisfies the constraint condition $\sum _{m=1}^{K}\,{\mu }_{m}=1$.

The accuracy of AD classification is verified by data obtained from ADNI (Alzheimer’s Disease Neuroimaging Initiative, http://adni.loni.usc.edu/) database. Ethical statements are present by ADNI, and we confirmed that all experiments were performed in accordance with relevant guidelines and regulations of the access. We also confirmed that all experimental protocols were approved by Sino-Dutch Biomedical & Information Engineering School, Northeastern University, China.

A total of 184 rs-fMRI data subjects were obtained, including 92 AD patients and 92 HC subjects. The details of the data are shown in Table 6.

**Table 6:** Details of ADNI data set.

Dataset	Subjects	Ages	slices	TE	TR
AD	92 [M49/F43]	73.6 ± 15	3.31 mm	30 ms	3.0 s
NC	92 [M30/f62]	75.1 ± 13	3.31 mm	30 ms	3.0 s

In order to verify the superiority of the proposed model and avoid the influence of classifiers, the classification performance was compared with RFE-GK by full frequency band network by the classifiers kernel Extreme Learning Machine (k-ELM)^32^ and kernel Support Vector Machine (k-SVM)^33^. The result is shown in the Table 7.

**Table 7:** Classification performance of two models.

Network Model	classifier	Accuracy %	Sensitivity %	Specificity %
RFE-GK^31^	k-SVM	67.5	75.0	60.0
	k-ELM	70.0	80.0	60.0
Multi-graph network	k-SVM	77.5	80.0	75.0
	k-ELM	82.5	85.0	80.0

It can be seen from the table that the classification performance of the proposed multi-graph models on the two classifiers is much better than the contrast model. The proposed model has 82.5% classification accuracy on the k-ELM and 77.5% classification accuracy on the k-SVM, while the contrast model has only 70% classification accuracy on the k-ELM and 67.5% classification precision on the k-SVM. It also can be seen that the classification effect of the two methods on k-ELM is better than that of k-SVM classification. The results show that the proposed multi-graph method can make full use of the hierarchical structure of the brain functional network, thus enabling better classifications of AD patients.

**Figure 12:**
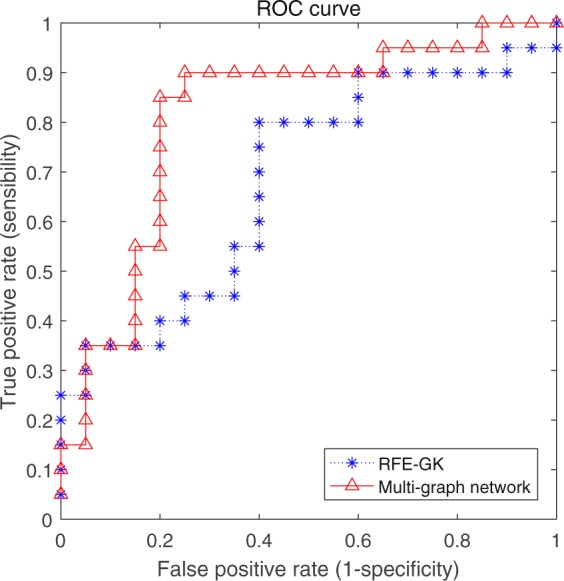
ROC curves of two models under k-ELM.

Second, the ROC curves are compared on k-ELM with better classification effect, shown in Fig. 12.

The superiority of the multi-graph method can be observed on the ROC curve of the above image, and the comparison between full frequency network model which has 0.68 AUC values and the multi-graph model having 0.81 AUC value through the experiment. It also shows that the brain network can be constructed from multi frequency bands angle using the multi-graph model, and the different frequency bands of brain activity are fully considered. The frequency domain information preserves the local topology of the brain network under different frequency bands, which fully integrates the brain activity information of the multi frequency band, and enhances the accuracy of the computer-aided diagnosis of Alzheimer’s disease. Thus, compared with the original full frequency model classification method, the proposed multi-graph model can be helpful to enhance the classification accuracy.

## Conclusion

A brain functional network model based on multi-graph is proposed in this paper. With the advantage of multi-graph, multi-band voxelwise brain network is constructed. As the possibility of the relationship between nodes can be described by multi-graph, a network model that can clearly describe the composition of brain function network is established. In this model, the components of the low frequency, intermediate frequency and high frequency bands in brain network are separated and analyzed, and some properties of them in the analysis of the brain network model are discussed. According to the results of network analysis, we can see that multi-graph model has important significance for the analysis and research of brain functional network. In our study, it is found that the distribution patterns of low-, intermediate- and high- frequency brain functional networks are of great significance to the study of the relationship between different cerebral regions.
